# Visible-light optical coherence tomography and its applications

**DOI:** 10.1117/1.NPh.12.2.020601

**Published:** 2025-04-09

**Authors:** Siyu Song, Tristan T. Hormel, Yali Jia

**Affiliations:** a Oregon Health and Science University, Casey Eye Institute, Portland, Oregon, United States; b Oregon Health and Science University, Department of Biomedical Engineering, Portland, Oregon, United States

**Keywords:** optical coherence tomography, optical coherence tomography angiography, spectroscopic analysis, retinal hemodynamics, retinal imaging, brain imaging

## Abstract

Visible-light optical coherence tomography (vis-OCT) is an emerging OCT technology that uses visible rather than near-infrared illumination and is useful for pre-clinical and clinical imaging. It provides one-micron level axial resolution and distinct scattering and absorption contrast that enables oximetry but requires additional considerations in system implementation and practical settings. We review the development of vis-OCT and demonstrated applications. We also provide insights into prospects and possible technological improvements that may address current challenges.

## 1 Introduction

Optical coherence tomography (OCT) is a noninvasive biomedical imaging technique that provides real-time, three-dimensional images at micron-scale resolution with several millimeter penetration depths. Since its initial development in the early 1990s,^1^^–^^4^ OCT has been applied to several clinical fields, such as ophthalmology,^5^ cardiology,^6^ dermatology,^7^ and dentistry.^8^ Due to the transparency of the eye, OCT achieves its greatest success in ophthalmology, and OCT devices for eye examinations are the only commercial OCT products that have become standard diagnostic tools in clinical settings. In the past decade, multiple functional extensions to the basic OCT approach were proposed. For example, Doppler OCT allows the observation of blood flow velocity,^9^ OCT angiography (OCTA) enables imaging of vasculature morphology without requiring intravenous dye for contrast,^10^^–^^12^ and optical coherence elastography provides information about tissue stiffness and elasticity.^13^

OCT systems can be categorized based on the wavelength of the light source. Most devices, including commercial devices, use conventional near-infrared (NIR) light, due to its excellent penetration depth and accessibility to available broadband light sources.^14^ OCT at the visible light band (vis-OCT) has garnered a lot of attention within the past few years. Its development has been driven by the recent introduction of commercial supercontinuum light sources. Compared with NIR-OCT, vis-OCT has intrinsic advantages in axial resolution due to its shorter central wavelength, which proportionally determines the resolution. Studies show that most NIR-OCT systems achieve 5- to 10-μm axial resolutions, while vis-OCT systems with a comparable bandwidth can provide sub-micron resolution.^15^^,^^16^ The high axial resolution allows vis-OCT to obtain more detailed visualization of retinal structures, including Bruch’s membrane,^17^ sub-layers of the inner plexiform layer (IPL),^18^ sub-bands in the photoreceptor outer segments,^19^ and the texture of nerve fibers.^20^ Thus, tissue abnormalities are able to be detected more efficiently.^21^ In addition, the higher scattering coefficients in biological tissue with visible light increase contrast in OCT imaging.^14^ Another main advantage of vis-OCT is the 100 times stronger hemoglobin absorption in the visible band than in the NIR band. This allows vis-OCT systems to quantify the total hemoglobin concentration^22^ and, more importantly, measure hemoglobin oxygen saturation through depth-resolved spectroscopic analysis.^23^^–^^25^ When incorporated with Doppler blood flow measurements, vis-OCT can measure the oxygen metabolism of the entire retinal circulation.^26^

To date, most vis-OCT systems are used for rodent retina imaging. For translation to human retinal imaging, there are more considerations including laser safety, patient comfort, and compliance during imaging.^21^ Some studies combined vis-OCT with other technologies such as adaptive optics (AO),^27^ scanning laser ophthalmoscopy (SLO),^27^ and autofluorescence imaging^28^ for more comprehensive information. Here, we review the development of vis-OCT and its applications.

## 2 System Construction

### 2.1 Broadband Visible Light Source

Standard spectral domain OCT (SD-OCT) systems employ compact super-luminescent diodes (SLDs) that generate smooth and stable spectra, but the center wavelengths of SLDs range from 670 to 1600 nm,^29^ which hardly covers the visible light range. External spectral broadening in special fibers, pumped by ultra-short laser pulses can meet this requirement.^30^ The first reported vis-OCT used a sub-15-fs Ti: sapphire laser to pump a photonic crystal fiber as the light source.^31^ The shaped emission spectrum ranged from 535 to 700 nm (centered at ∼600  nm), reaching 0.9  μm axial resolution in air. However, it was based on a time domain configuration, which did not allow for *in vivo* imaging due to speed limitations. Moreover, this external broadening approach results in power fluctuations, spectral modulation, and more quantum shot noise.^32^^,^^33^ Another method generated visible light by focusing a Ti: sapphire laser through a frequency-doubling crystal.^34^ Although the crystal offered great power stability, the bandwidth was only ∼10  nm which limited the axial resolution.

Supercontinuum light sources that cover wavelengths from 450 to 2400 nm can be combined with bandpass filters to provide visible light; more and more studies are doing so.^23^^,^^35^^–^^37^ Most commercially available supercontinuum sources generate broadband light by launching a long light pulse (picosecond or nanosecond) through a nonlinear optical fiber.^38^ In the regime of long pulses, the initial broadening of the supercontinuum is produced by nonlinear amplification of quantum noise and the resulting spectra are thus particularly noisy and uncorrelated from pulse-to-pulse.^38^^,^^39^ Consequently, supercontinuum sources exhibit higher noise levels, as opposed to SLDs. But rather than being a liability, excess noise can be applied for spectrometer characterization and cross-calibration. Kho et al.^40^ demonstrated its utility by improving the spectral resolution in vis-OCT system and visualized a hyporeflective band inner to the external limiting membrane in the mouse retina. Besides, there are some ways to mitigate the effects of relative intensity noise (RIN) and excess noise. The first is to increase the repetition rate of the supercontinuum sources.^41^^,^^42^ Current state-of-the-art vis-OCT systems use high repetition rates up to 320 MHz.^43^ The second is to improve signal detection methods. For instance, several studies validated balanced detection as an effective way to achieve low-noise properties.^44^^–^^46^ The third is to use all-normal-dispersion (ANDi) fibers to generate supercontinuum light.^38^^,^^47^^,^^48^ The representatives include shot-noise limited SD-OCT with a supercontinuum source generated by an ANDi fiber and a femtosecond laser, which enhanced the image quality in terms of both contrast and sensitivity.^38^

Most low-noise supercontinuum sources with high repetition rates are expensive.^44^ To reduce the cost of vis-OCT, some groups explored the possibility of achieving a broad visible spectral range with cheaper alternatives. Lichtenegger et al.^49^ combined two discrete visible spectral windows generated by two SLDs with a deep learning network to reconstruct a high-resolution image equivalent to that with a continuous spectral band. In addition, recently, Gupta et al.^50^ demonstrated a three-SLD combined source of vis-OCT for human retinal imaging. Another feasible way to reduce costs is to use low-repetition-rate supercontinuum sources and balanced detection methods. One example is that Kho et al.^44^ used a 30-MHz repetition rate supercontinuum laser with precise balancing, providing comparable performance that requires 5.2 times repetition rate without balancing, achieving a cost reduction of $30 k.

### 2.2 System Implementation

**Fig. 1:**
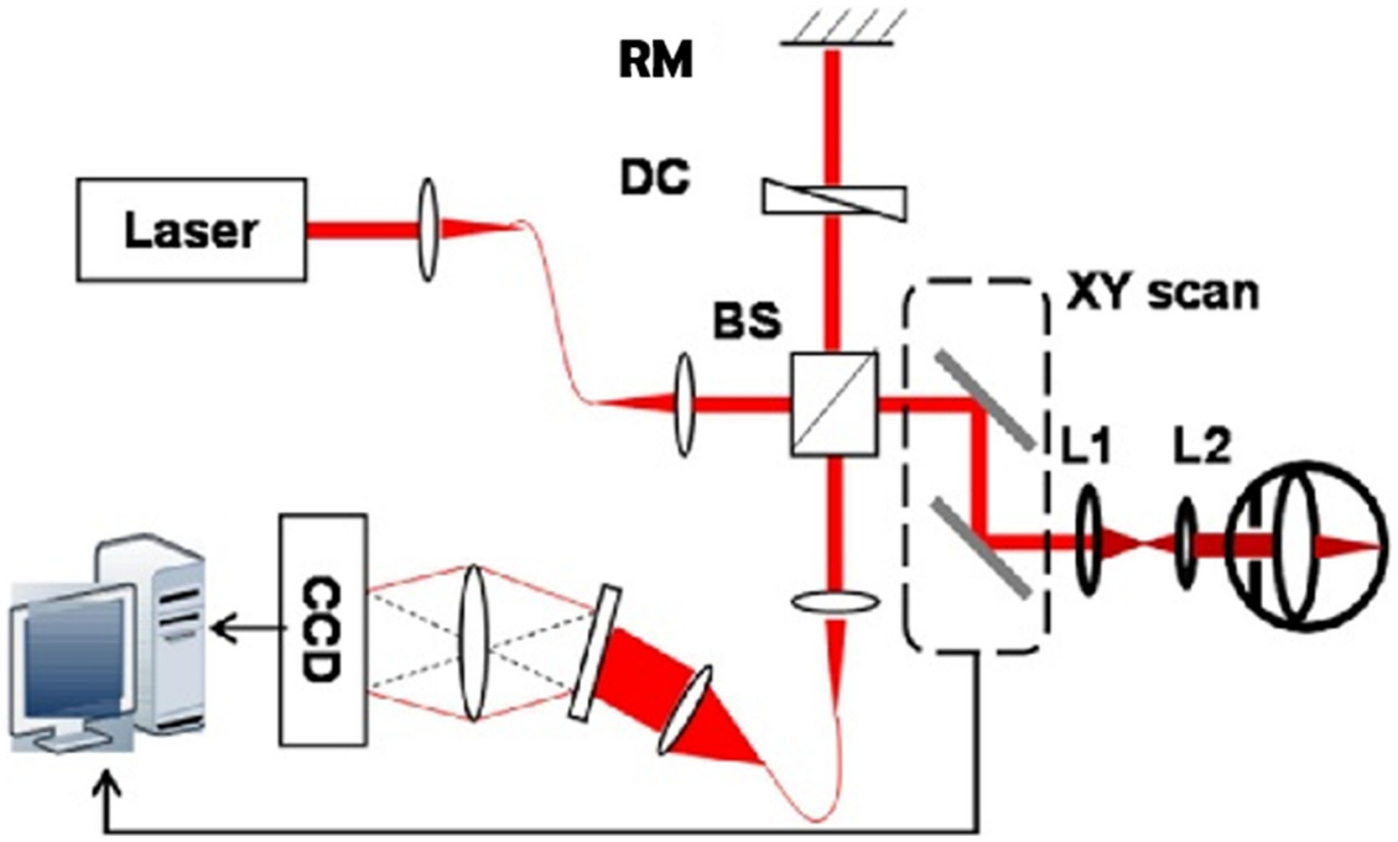
Free-space vis-OCT system. Lens L1 and L2 relay the beam onto the sample. BS, beam splitter; RM, reference mirror; DC, dispersion control. Reproduced with permission from Ref. 23.

Due to the available visible light sources, contemporary vis-OCT systems are built on the SD-OCT configuration, and both free-space and fiber-based Michelson interferometers have been reported. Most free-space configurations use beam splitters to separate light into the sample and reference arms which can avoid power loss and imbalanced dispersion in the fiber couplers.^20^^,^^22^^,^^23^^,^^51^ A representative free-space system layout is illustrated in Fig. 1.^23^ This system uses point scanning, but this is not the only option. Robles et al.^52^ developed a line-field vis-OCT system using a cylindrical lens and Scholler et al.^53^ reported a full-field vis-OCT system with a light-emitting diode (LED) light source.

**Fig. 2:**
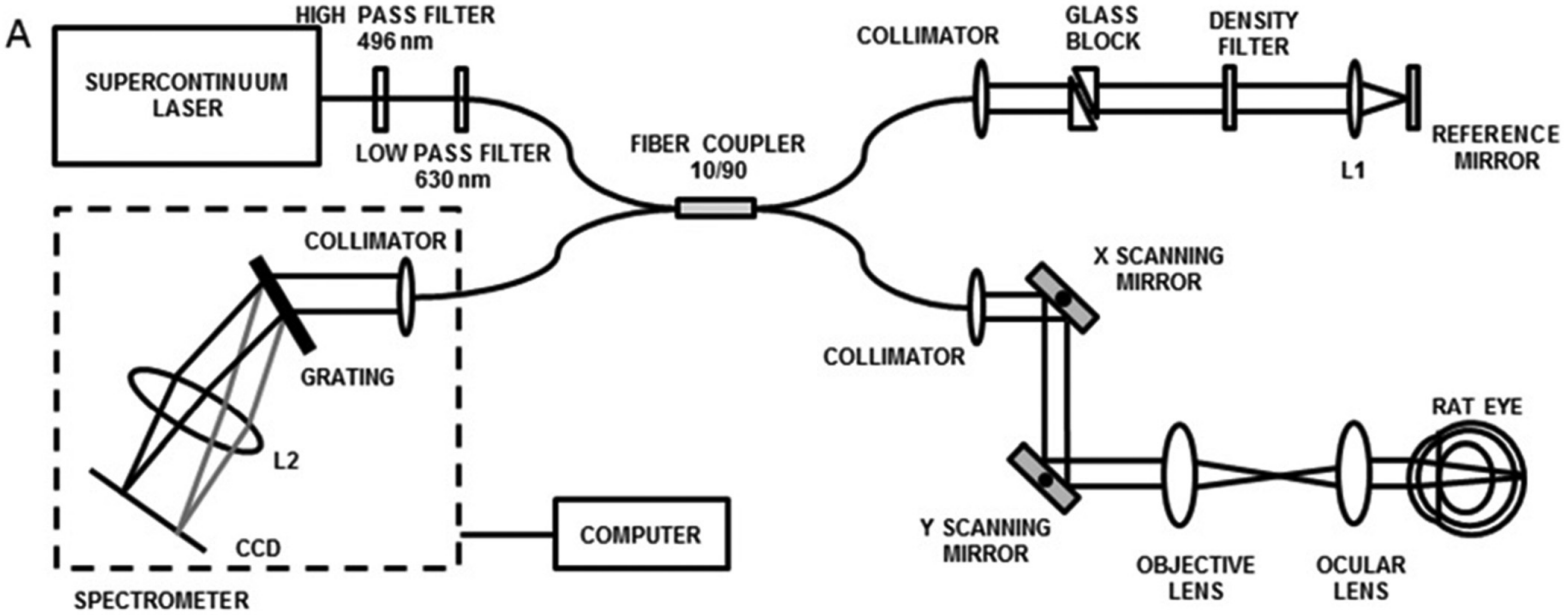
Fiber-based vis-OCT system. The objective lens and ocular lens relay the beam onto the sample. A pair of triangular glass blocks compensates for dispersion mismatch. A neutral density filter prevents the saturation of the camera sensor. Reproduced with permission from Ref. 16.

Another type is fiber-based implementation, using a fiber coupler to separate light (Fig. 2).^16^^,^^42^^,^^54^^,^^55^ It should be noted that for both free-space and fiber-based systems, an unbalanced splitting ratio (e.g., 10/90 or 25/75) is preferred for maximizing interference signals.^42^

Several aspects should be considered in vis-OCT system design:

(1) Transverse optical resolution. It is inversely proportional to the numerical aperture of the optics in the sample arm and can be worsened by chromatic aberrations which arise from variations in refractive index and are more pronounced at shorter wavelengths. To mitigate these effects, achromatic lenses made from low-dispersion materials are commonly employed. For instance, Chong et al.^17^ used a zero-power triplet achromatizing lens in the sample arm and a focusing lens chosen for a linear chromatic focal shift within the spectrometer to minimize longitudinal chromatic aberration. In addition, Song et al.^56^ used a moving averaging algorithm to minimize transverse chromatic aberration. Both approaches can improve OCT images with greater clarity. When used for eye imaging, the tear film, cornea, and intraocular lens introduce additional optical aberrations, which can be further corrected with AO.^27^^,^^57^
(2) Imaging depth. The optical attenuation of biological tissues is strong in the visible range, presenting a constraint to imaging beyond a few hundred micrometers. In theory, the maximum imaging depth is given by the Nyquist criterion as
  $$z_{\max}=\frac{\lambda_{0}^{2}N_{p}}{4n\Delta \lambda },$$ (1)
  where $N_{p}$ is the pixel number of the detector array. Therefore, spectrometers should be designed to use as many of the available camera pixels as possible. If the sample strays over the zero-path length border, it begins to overlap with its mirror image because the Fourier transform leads to the complex conjugate artifact. Several methods can be used to remove this artifact, including Hilbert phase microscopy,^58^ off-axis holography,^59^ and phase-shifting interferometry.^60^ Maximum imaging depth is doubled in this way. However, sensitivity roll-off caused by low-pass filtering of finite sampling elements further limits the attainable depth range.^61^ In addition, this effect can be exacerbated by unequal spectral bandwidths of the pixel integrals of the detector array.^62^ Recently, Wang et al.^63^ developed the first linear-in-k vis-OCT spectrometer to decrease roll-off. Combined with reference pathlength modulation to expand the imaging depth, a 7.2-dB roll-off over a full range of 1.74 mm was achieved. On top of that, there is only a small range within the whole depth that can resolve details with high clarity, which is referred to as depth of focus. For conventional OCT systems, Gaussian beams are used to illuminate the sample, and the depth of focus is therefore inversely proportional to transverse resolution. In practice, OCT systems should be designed to balance this trade-off based on applications.
(3) Field of view (FOV). Compared with NIR light, visible light is more susceptible to chromatic aberrations and dispersion in optical components and tissue, which can degrade the image quality, making it harder to achieve a clear and wide FOV. Therefore, more sophisticated optical designs, or integrations with other technologies, are required to alleviate these effects. For instance, montaging can be used to expand FOV, which involves multiple smaller scan acquisition, image alignment, and stitching.^57^^,^^64^

The specifications of several recently developed NIR-OCT and vis-OCT systems are summarized in Table 1.

**Table 1:** Summary of representative NIR- and vis-OCT systems developed recently.

Wavelength range	NIR			Visible		
Authors	Liang et al.^65^	Ni et al.^66^	Neuhaus et al.^67^	Wang et al.^63^	Xu et al.^68^	Revin et al.^69^
Year	2024	2024	2024	2024	2024	2023
Light source	Swept-source	Swept-source	Super-luminescent diode	Supercontinuum source	Supercontinuum source	Supercontinuum source
Center wavelength	1060 nm	1060 nm	840 nm	575 nm	620 nm	585 nm
Bandwidth	100 nm	100 nm	50 nm	150 nm	118 nm	270 nm
Axial resolution	5 μm (in air)	7 μm (in air)	1.7 μm (in air)	1.3 μm (in water)	1.4 μm (in air)	1.58 μm (in air)
Transverse resolution	8.9 μm (in tissue)	46.4 μm (in tissue)	6.3 μm (in air)	—	4.5 μm (in air)	6.9 μm (in air)
Imaging depth	—	—	—	1.74 mm (in water)	—	0.85 mm (in air)
Sensitivity	—	—	70 dB	90.5 dB	—	75 dB
Roll-off	—	—	3 dB/mm	4.14 dB/mm	—	—
FOV	112 deg	140 deg	—	60 deg	—	8 mm × 8 mm
A-line rate	400 kHz	800 kHz	2.5 MHz	120 kHz	62.5 kHz	125 kHz
Sample	Rat retina	Infant retina	Human cornea	Human retina	Mouse brain	Human skin

### 2.3 Integration with Other Technologies

**Fig. 3:**
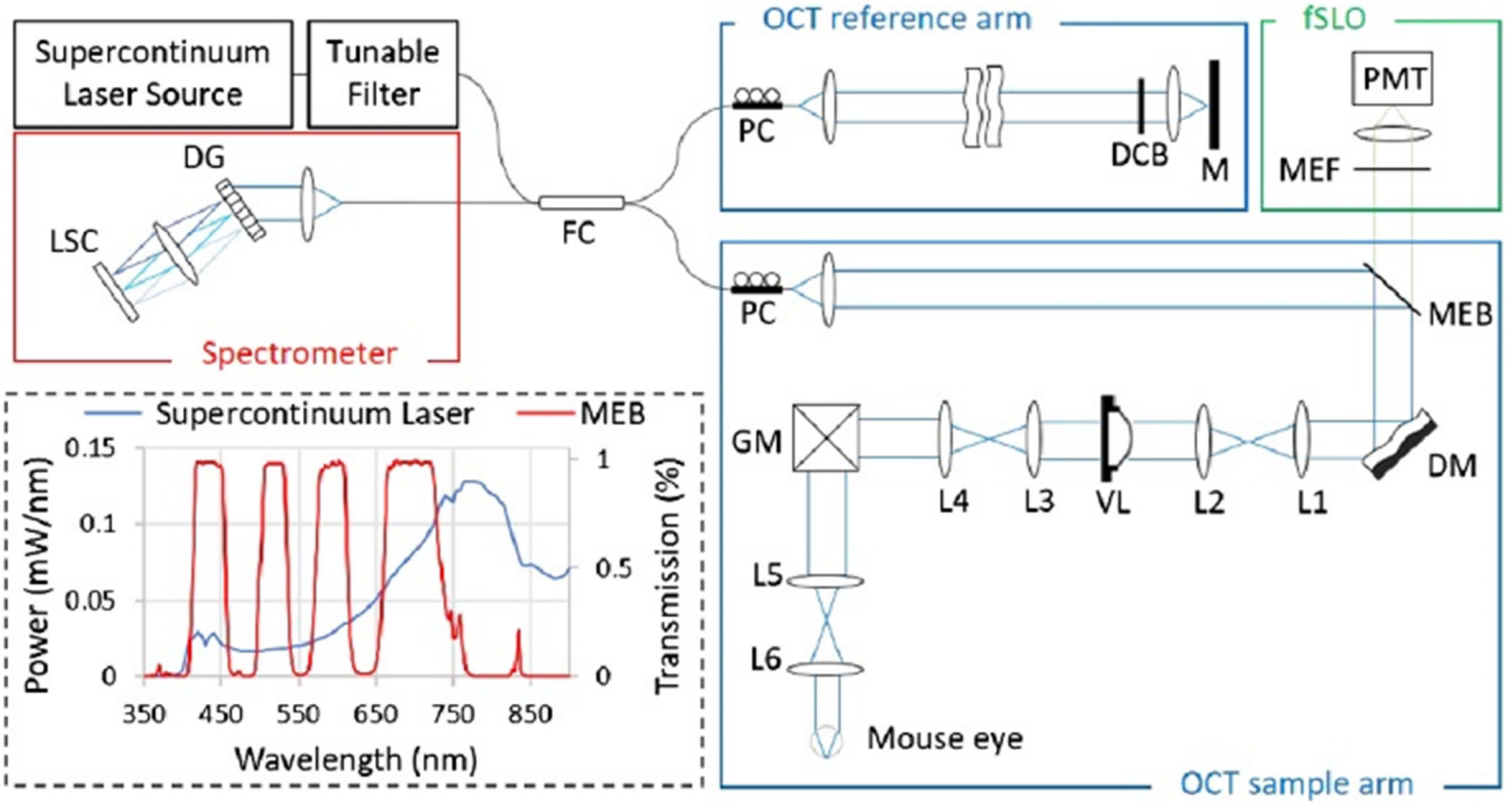
Multimodal AO-vis-OCT and SLO system. In the OCT sample arm, a deformable mirror (DM) is used for aberration correction, and a variable focus lens (VL) is used to control the focal plane in the sample. The transmission rate of the multi-edge filter beam splitter (MEB) varies with wavelength, making back-scattered OCT signal collected by the spectrometer and fluorescence emission signal transmitted through to a photo-multiplier tube (PMT). In the SLO module, a multi-edge filter (MEF) is used to reject any residual excitation light before PMT. Reproduced with permission from Ref. 27.

vis-OCT has been combined with AO^27^ and SLO.^28^^,^^70^^,^^71^ AO can help to correct optical aberrations and approach diffraction-limited imaging. In addition, SLO enables endogenous and exogenous fluorescence signal imaging which can visualize and differentiate cell types^72^^,^^73^ and may provide complementary information to the analysis of the pathology.^74^ A schematic of the multimodal AO-vis-OCT and SLO system is shown in Fig. 3.^27^

In this system, the light was tuned to a center wavelength of 470 nm with a bandwidth of 30 nm, which is compatible with the excitation of enhanced green fluorescent protein (EGFP). A mouse with EGFP-labeled ganglion cells was imaged. After AO correction, the brightness of cross-sectional OCT images increased (∼1.5  dB), and the ganglion dendrites were clearer. Similarly, Jiang et al.^37^ used a single broadband light source centered at 480 nm for autofluorescence imaging, whose signals primarily originate from the accumulation of lipofuscin within the retinal pigment epithelium (RPE). They successfully demonstrated lipofuscin accumulation in the RPE with aging.

**Fig. 4:**
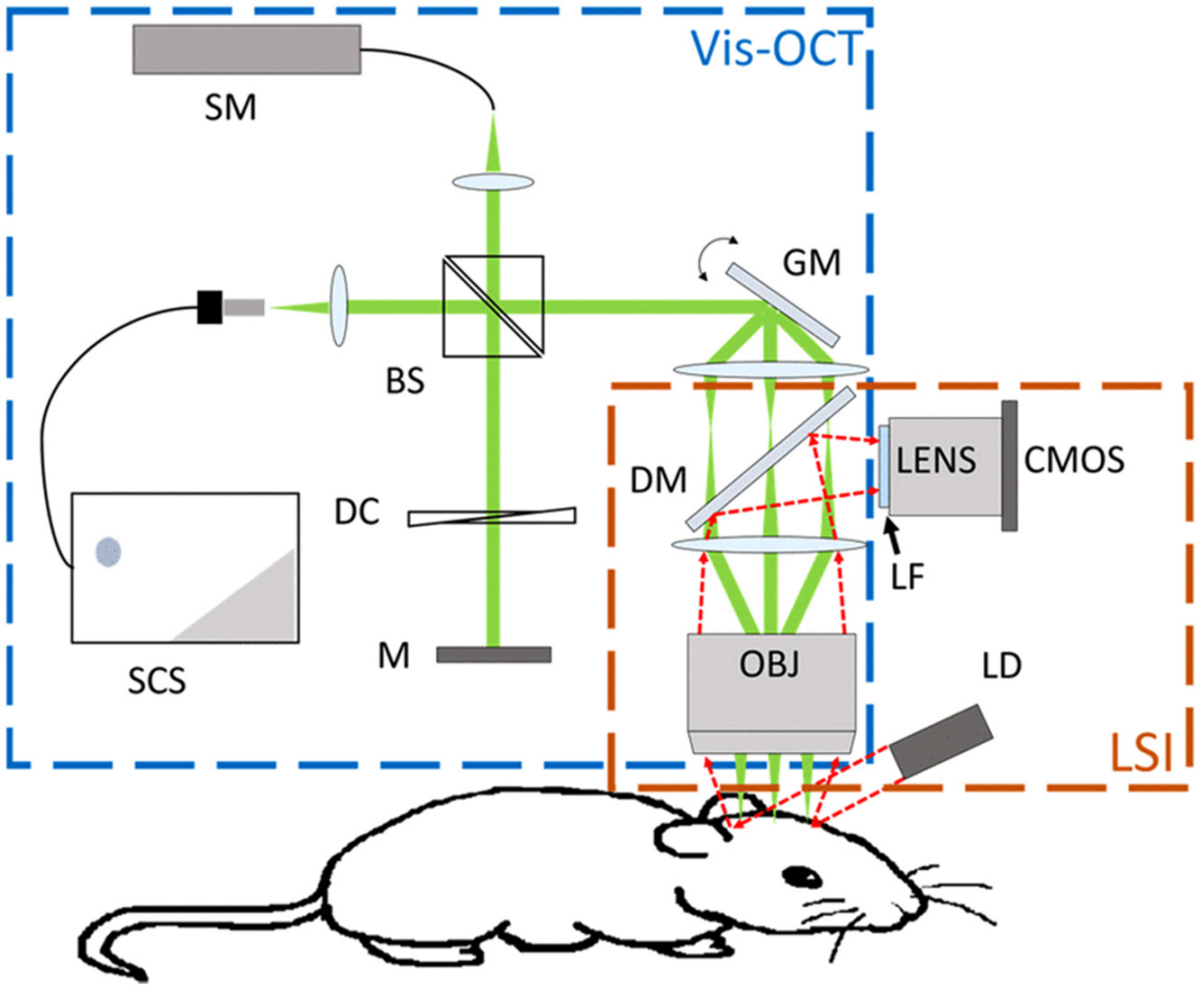
Vis-OCT and LSI system. In the OCT module, a 4f telescope system is added between galvanometer mirrors (GM) and an objective lens (OBJ), accommodating the LSI light path. In the LSI module, a laser diode (LD) is used to illuminate the cranial window of the mouse. The scattered light is collected by the OBJ and then passes through the first lens of the 4f telescope system, a dichroic mirror (DM), and a long-pass filter (LF), captured by a CMOS camera. Reproduced with permission from Ref. 75.

Another technology compatible with vis-OCT is laser speckle imaging (LSI),^75^ as indicated in Fig. 4. In this study, the aim was to measure changes in cerebral blood flow (CBF), vascular morphology, and oxygen saturation of a distal middle cerebral artery occlusion (dMCAO) mouse model. LSI offered full-field, real-time imaging of dynamic CBF changes before, during, and after stroke and provided large FOV to guide vis-OCT to locate the area of interest for oxygen saturation and angiography analysis. LSI was also able to provide relative CBF changes on the superficial tissue layer, which could be used as a criterion for a successful stroke.

## 3 Advanced Functional and Structural Modalities

### 3.1 vis-OCT Oximetry

OCT combined with spectroscopic analysis can be used to assess localized absorption spectra of endogenous or exogenous chromophores in biological tissue,^76^ and the spectral features can provide information on the composition and function of normal or pathological regions, specifically imaging endogenous chromophores such as especially hemoglobin.^14^ Attempts to measure blood oxygenation using OCT date back almost two decades,^77^^–^^79^ in which the spectral contrast between oxygenated hemoglobin (${\mathrm{HbO}}_{2}$) and deoxygenated hemoglobin (Hb) was characterized near 800 nm. However, these approaches have limited accuracy because the extinction due to light absorption in the NIR is overwhelmed by scattering.^80^ In the early 2010s, Robles et al.^36^ and Yi et al.^81^ explored the possibility of quantifying oxygen saturation (${\mathrm{sO}}_{2}$) with vis-OCT. This method can provide several advantages. First, the absorption characteristics of ${\mathrm{HbO}}_{2}$ and Hb have much better contrast for spectroscopic analysis in the visible range, showing peaks that can be distinguished in the logarithm of the extinction coefficient curve (${\mathrm{HbO}}_{2}$: 540 and 575 nm, Hb: 555 nm).^80^ Second, the extinction coefficients of ${\mathrm{HbO}}_{2}$ and Hb are two orders of magnitude stronger in the visible range than in the NIR.^80^ Third, compared with the NIR range (especially above 940 nm), water absorption is much lower in the visible spectrum, allowing for more direct measurement of hemoglobin’s oxygen-dependent absorption characteristics.^82^ Further explorations include *in vivo* measurement of blood oxygenation,^52^ and the first demonstration of *in vivo* retinal oximetry in rodents.^23^ Later, more studies used this technology to monitor how progressive hypoxic challenge and intraocular pressure (IOP) elevation affect retinal oxygen metabolism.^26^^,^^83^^–^^85^ In addition, advances in automated detection of posterior vascular boundaries,^80^ acquisition of multiple circumpapillary scans,^86^ and quantitative quality-control metrics^87^ have also contributed to the development of retinal oximetry in major vessels.

To extract hemoglobin content, spectral interferograms are first processed by standard OCT signal processing, including background subtraction, dispersion compensation, and k-space resampling. Then, vessel boundaries are determined from the reconstructed OCT B-scan images and *en face* images. Assuming the concentrations of ${\mathrm{HbO}}_{2}$ and Hb, noted by $C_{{\mathrm{HbO}}_{2}}$ and $C_{\mathrm{Hb}}$, are uniform in one single vessel, the wavelength-dependent A-line reflectance intensity I(z,λ) depends on depth z and incident wavelength λ as^80^

$$I(z,\lambda )=I_{0}(\lambda )R_{0}r(\lambda )e^{-2(z-z_{0})[C_{{\mathrm{HbO}}_{2}}E_{{\mathrm{HbO}}_{2}}(\lambda )+C_{\mathrm{Hb}}E_{\mathrm{Hb}}(\lambda )]},$$ (2)

where $z_{0}$ is the depth of the vessel’s anterior boundary, $I_{0}(\lambda )$ is the incident spectrum, $R_{0}$ is the reference arm reflectance, $E_{{\mathrm{HbO}}_{2}}(\lambda )$ and $E_{\mathrm{Hb}}(\lambda )$ are the effective extinction coefficients of ${\mathrm{HbO}}_{2}$ and Hb, and r(λ) is the reflectance at the posterior vessel boundary, which can be modeled as a power law under the first-order Born approximation,^23^

$$r(\lambda )=A\lambda^{-\alpha },$$ (3)

where A is a constant^88^ and the value of α depends on the tissue type and has been characterized to be ∼1 with variations around ±0.2.^89^^,^^90^ The optical density (OD) can then be obtained as

$$\mathrm{OD}(z,\lambda )=\ln(\frac{I(z,\lambda )}{I_{0}(\lambda )})=-2(z-z_{0})[C_{{\mathrm{HbO}}_{2}}E_{{\mathrm{HbO}}_{2}}(\lambda )+C_{\mathrm{Hb}}E_{\mathrm{Hb}}(\lambda )]-\alpha \;\ln(\lambda )+\ln(AR_{0}),$$ (4)

which can be measured using a series of short-time Fourier transforms to the interference spectrum.^91^ Here, several Gaussian window groups are applied, leading to a few split spectra. To perform spectroscopic fitting, the linear equation for OD is rewritten as a matrix form with a size of n, where n is the number of split spectra

$$[\begin{array}{c} \mathrm{OD}(z,\lambda_{1})\\ \mathrm{OD}(z,\lambda_{2})\\ \begin{array}{c} \ldots \\ \mathrm{OD}(z,\lambda_{n}) \end{array} \end{array}]=[\begin{array}{cc} E_{{\mathrm{HbO}}_{2}}(\lambda_{1}) & \begin{array}{ccc} E_{\mathrm{Hb}}(\lambda_{1}) & \ln(\lambda_{1}) & 1 \end{array}\\ E_{{\mathrm{HbO}}_{2}}(\lambda_{2}) & \begin{array}{ccc} E_{\mathrm{Hb}}(\lambda_{2}) & \ln(\lambda_{2}) & 1 \end{array}\\ \begin{array}{c} \ldots \\ E_{{\mathrm{HbO}}_{2}}(\lambda_{n}) \end{array} & \begin{array}{c} \begin{array}{ccc} \ldots & \ldots & \ldots \end{array}\\ \begin{array}{ccc} E_{\mathrm{Hb}}(\lambda_{n}) & \ln(\lambda_{n}) & 1 \end{array} \end{array} \end{array}][\begin{array}{c} -2(z-z_{0})C_{{\mathrm{HbO}}_{2}}\\ -2(z-z_{0})C_{\mathrm{Hb}}\\ \begin{array}{c} -\alpha \\ \ln(AR_{0}) \end{array} \end{array}].$$ (5)

According to the Kramers–Kronig relations hemoglobin absorption affects the optical scattering of blood, resulting in an oxygenation-dependent optical scattering spectrum. Thus, the extinction coefficients $E_{{\mathrm{HbO}}_{2}}(\lambda )$ and $E_{\mathrm{Hb}}(\lambda )$ combines both absorptions ($E_{a}$) and scattering contributions ($E_{s}$) as

$$E=E_{a}+WE_{s},$$ (6)

where W is the blood cell packing factor that weights the scattering spectrum, suggested to be set between 0.2 and 0.4.^92^ For whole blood in retinal vessels, the specific values of $E_{a}$ and $E_{s}$ can be found in the literature^93^ and are wavelength-dependent. Assuming the second matrix on the right-hand side of Eq. (5) is M,

$$M=[\begin{array}{c} -2(z-z_{0})C_{{\mathrm{HbO}}_{2}}\\ -2(z-z_{0})C_{\mathrm{Hb}}\\ \begin{array}{c} -\alpha \\ \ln(AR_{0}) \end{array} \end{array}].$$ (7)

When other parameters in Eq. (5) are confirmed, it can be fit with a non-negative least-square fitting model, and ${\mathrm{sO}}_{2}$ is calculated as the ratio of oxygenated hemoglobin to the total hemoglobin concentration:

$${\mathrm{sO}}_{2}=\frac{C_{{\mathrm{HbO}}_{2}}}{C_{{\mathrm{HbO}}_{2}}+C_{\mathrm{Hb}}}=\frac{M(1)}{M(1)+M(2)}.$$ (8)

**Fig. 5:**
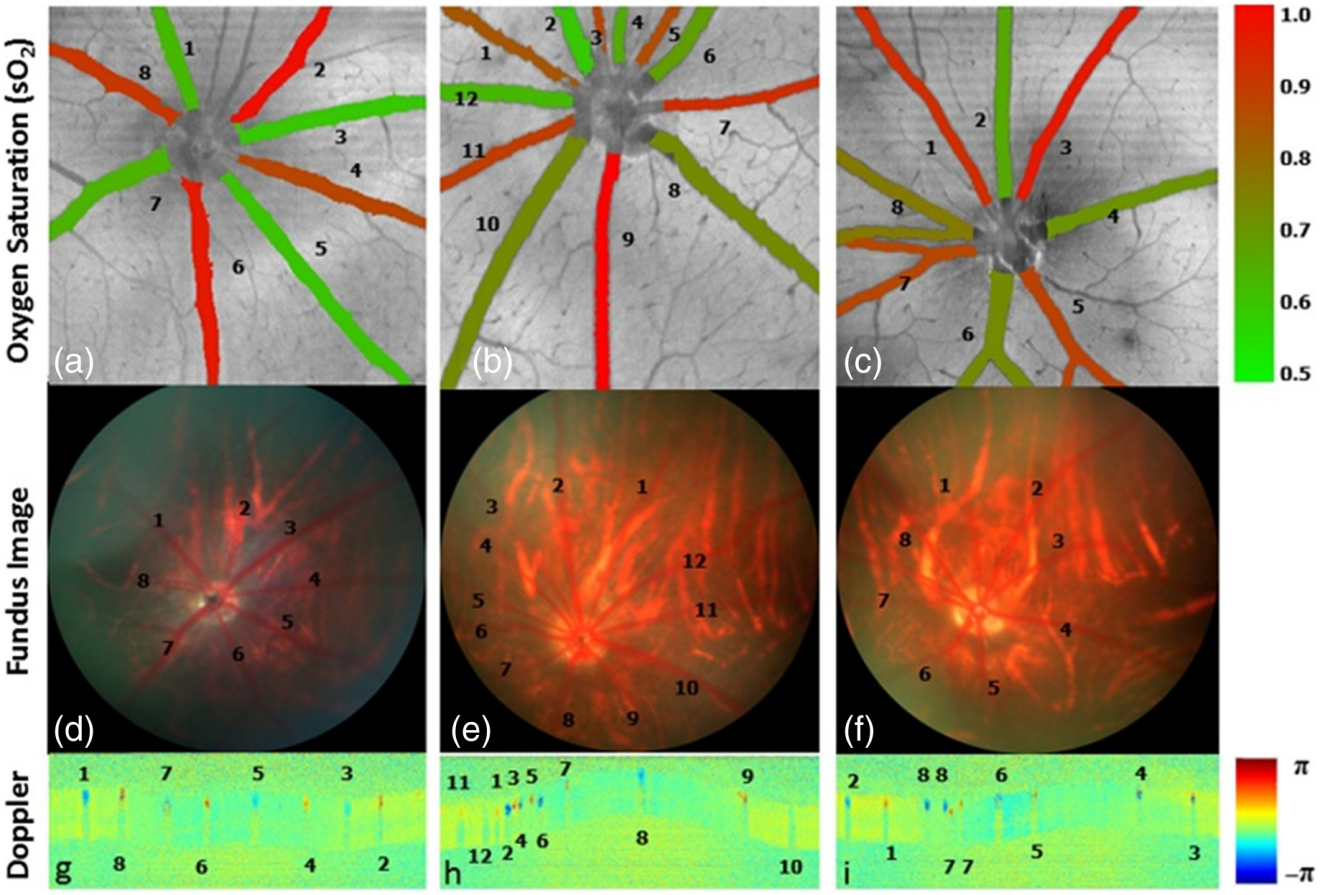
Rat retina arteriovenous identification. (a)–(c) Arteries demonstrate higher ${\mathrm{sO}}_{2}$ (red color) than veins (green color) under vis-OCT oximetry. The ${\mathrm{sO}}_{2}$ of each vessel is overlaid on structural *en face* images. This identification is supported by their morphologic appearance on fundus images in panels (d)–(f), where arteries tend to have a smaller diameter, stronger reflection, and lighter color than veins. (g)–(i) Another support from Doppler OCT, with arterial flow noted to be away from the optic disc, creating a positive Doppler shift (red color) and veins flowing toward the disc, with a negative shift (blue color). Reproduced with permission from Ref. 80.

For healthy subjects, arteries have higher ${\mathrm{sO}}_{2}$ than veins,^23^^,^^25^ and this can be used to distinguish these major vessels. Apart from that, the identification of arteries and veins can also be verified by Doppler OCT, with arterial blood flow away from the optic disc producing a positive Doppler shift, and venous blood flow toward the optic disc producing a negative Doppler shift. Deep-learning methods also enable the classification with high accuracy.^94^ A demonstration of ${\mathrm{sO}}_{2}$ within major vessels is shown in Fig. 5.^80^ Alterations in ${\mathrm{sO}}_{2}$ are believed to be a biomarker for ocular diseases,^95^ including diabetic retinopathy, central retinal vein occlusion, retinitis pigmentosa, and glaucoma.^96^^,^^97^

Other than the least-squares fitting method, Liu et al.^98^ demonstrated a data-driven approach, termed deep spectral learning, to achieve oximetry. In that study, they trained two neural network models to link the spectral measurements and the independently measured pulse oximeter labels. The predicted ${\mathrm{sO}}_{2}$ showed lower mean-square errors than those of the least-squares fitting approach (<1/3).

### 3.2 vis-OCT Angiography

OCTA, one of the primary functional extensions of OCT, enables 3D visualization of blood flow within vascular networks. The principle of OCTA is to utilize the differences between successive OCT scans at the same location, which arise from the movement of particles such as red blood cells, to construct a contrast signal.^99^ It can image individual vascular plexuses throughout the retina and choroid and help with the identification of abnormal vascular and blood flow changes in ocular diseases, such as glaucoma,^100^^,^^101^ age-related macular degeneration (AMD),^102^ and diabetic retinopathy.^103^^,^^104^ Multiple algorithms have been used for vis-OCTA. For example, Pi et al.^25^ used a split-spectrum amplitude-decorrelation angiography (SSADA) algorithm to produce 3D OCTA signals. To put it simply, SSADA splits the spectrum into S bands and calculates the decorrelation value of each band among consecutive B-scans at the same location.^105^ Given that N is the repeated B-scans, and $A_{i,s}$ is the A-scan generated by the $s^{\mathrm{th}}$ split spectrum corresponding to the $i^{\mathrm{th}}$ B-scan at the same position, the final angiography D is obtained by

$$D=1-\frac{1}{S(N-1)}\overset{N-1}{\underset{i=1}{\sum }}\overset{S}{\underset{s=1}{\sum }}\frac{2A_{i,s}A_{i+1,s}}{A_{i,s}^{2}+A_{i+1,s}^{2}}.$$ (9)

**Fig. 6:**
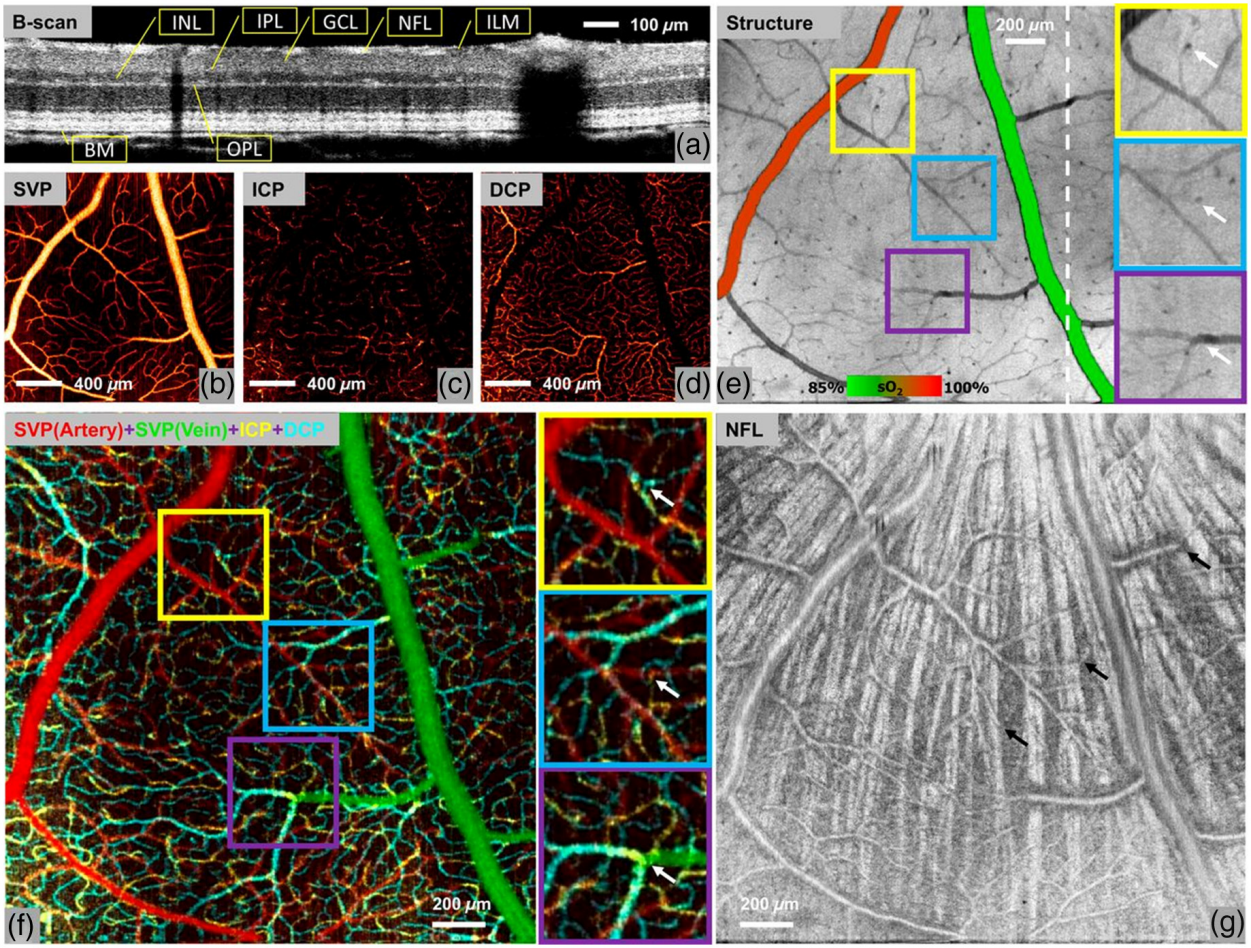
vis-OCT structural images and angiography images of a brown Norway rat retina. (a) B-scan image of the rat retina using vis-OCT. (b)–(d) *En face* images of vascular/capillary plexuses. SVP projected in the NFL and GCL slabs. ICP projected in the slab containing the inner border of the INL. DCP projected in the slab containing the outer border of the INL. (e) *En face* image projected from the ILM to BM, overlaid with measured oxygen saturation (${\mathrm{sO}}_{2}$) values in major vessels to differentiate arteries from veins in an animal breathing 100% $O_{2}$. Inter-plexus capillaries (white arrows) appear as dark spots due to greater light absorption than neighboring capillaries. (f) Overlaid *en face* angiograms of three vascular/capillary plexuses to demonstrate the detailed organization of the retinal circulation. Examples of inter-plexus capillaries (indicated by white arrows in the enlarged images) were validated by observing their presence in corresponding locations. (g) *En face* projection of the NFL slab. The SVP was found to run anterior to the nerve fiber bundles (bright radial striations), which appear posterior to the vessels. The inter-plexus capillaries (black arrows) penetrate between NFL bundles and connect the SVP to the ICP and DCP. Reproduced with permission from Ref. 25.

The result is shown in Fig. 6, demonstrating the detailed organization of the retinal circulation. To simplify OCTA data processing on retinal vasculature segmentation, Guo et al.^106^ developed a deep-learning-based method to segment vessels in the superficial vascular plexus, intermediate capillary plexus, and deep capillary plexus directly from volumetric OCTA data, effectively reducing errors caused by manual processing.

In addition, when OCTA is used in conjunction with oximetry, it offers insight into oxygen levels of capillaries within a specific tissue layer or reveals regional metabolic variations.^25^^,^^107^^,^^108^ The ${\mathrm{sO}}_{2}$ could be calculated directly from wavelength-dependent OCTA signals without the need for cumbersome image processing to localize vessels.^24^ A similar strategy was adapted for both exposed cortical imaging and optical-power-canceled retinal imaging in rodents.^22^ And Chen et al.^24^ expanded the theoretical model by taking blood flow contrast into account in OCTA-based oximetry. Using this technology to determine 3D capillary-level maps of oxygenation can help to clarify and expand the understanding of retinal oxygen supply in health and disease states.

### 3.3 vis-OCT Fibergraphy

vis-OCT fibergraphy (vis-OCTF) is used to visualize and quantify retinal ganglion cell (RGC) axon bundles. To extract the fibergram, the first step is to use a segmentation algorithm to isolate the retinal nerve fiber layer (RNFL) from OCT volume. Then, short-time Fourier transforms are performed to take advantage of the strong backscattering nature of the RGC axon bundles, and the mean intensity spectrum within the RNFL region is calculated and subtracted at each pixel. Third, summing this difference across the spectrum to yield a single difference from the mean (DiFM) value for each pixel, and pixels with a higher DiFM indicate higher backscattering than surrounding tissues. The process can be expressed as^55^^,^^109^^,^^110^

$$\mathrm{DiFM}(x,y,z)=\overset{\lambda_{n}}{\underset{\lambda =1}{\sum }}[{\overset{¯}{I}}_{\mathrm{RNFL}}(\lambda )-I(x,y,z,\lambda )],$$ (10)

where ${\overset{¯}{I}}_{\mathrm{RNFL}}(\lambda )$ is the mean intensity and I(x,y,z,λ) is the intensity of a voxel for a given wavelength, respectively. In the end, create a 3D binary mask based on the DiFM image and multiply it with the segmented RNFL B-scans, and the *en face* image, namely fibergram, is generated by averaging the 3D volume along the depth direction.

**Fig. 7:**
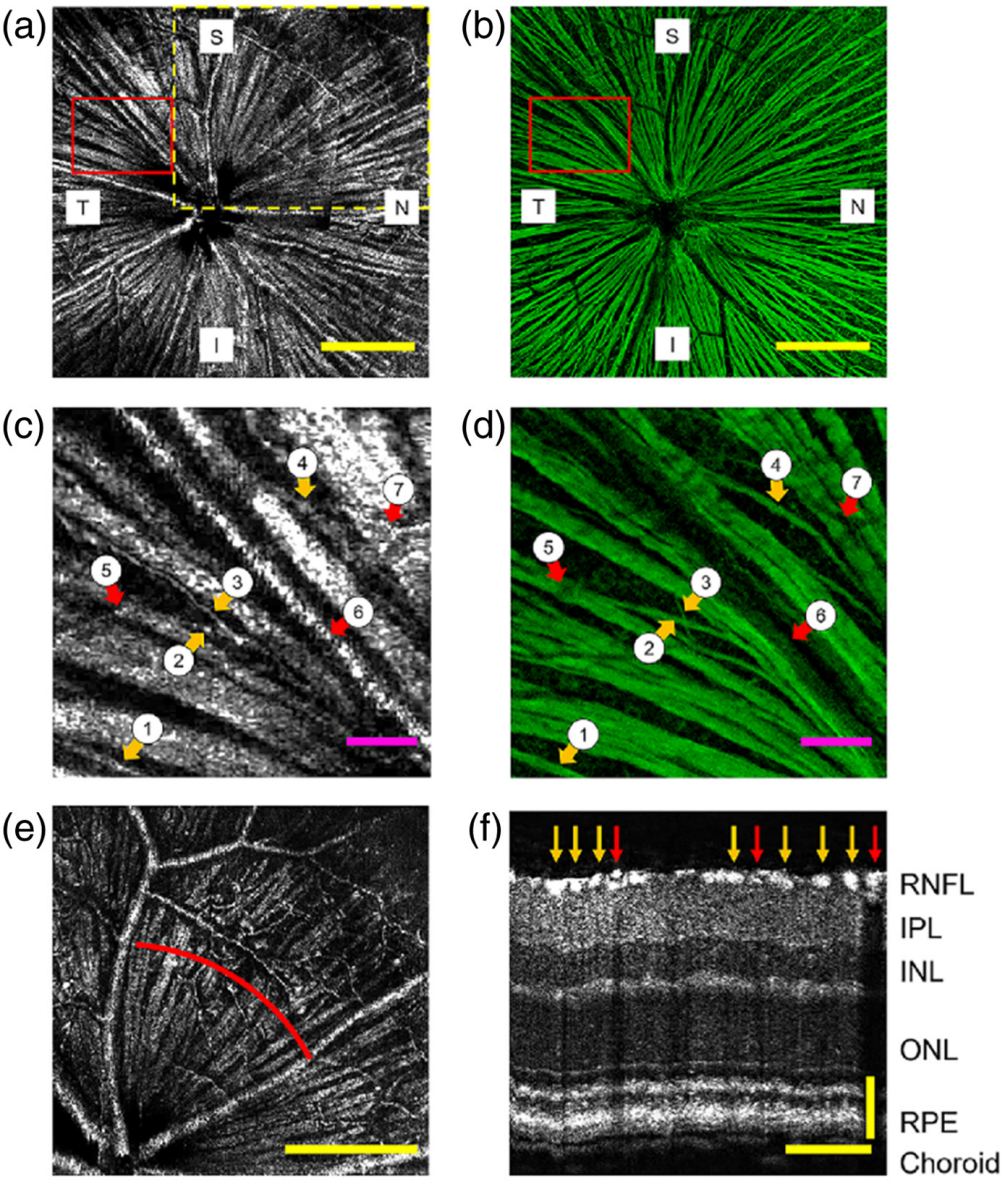
Comparing vis-OCTF and confocal microscopy of RGC axon bundle images. (a) *In vivo* vis-OCT fiber gram. (b) *Ex vivo* confocal microscopy image of the immunostained flat-mounted retina. (c) Magnified view of the highlighted area in panel (a). (d) Magnified view of highlighted area in panel (b). The orange arrows (1 to 4) indicate four small RGC axon bundles visible in both vis-OCT fibergrams and confocal microscopy images. The red arrows (5 to 7) indicate blood vessels. (e) Fibergram from a single OCT volume indicated by the dashed yellow box in panel (a). (f) B-scan image reconstructed from red arc shown in panel (e) centered 400  μm from the ONH. The orange arrow indicates RGC axon bundles (bright spots), and the red arrows indicate blood vessels (dark shadows through all subsequent layers). Reproduced with permission from Ref. 55.

To demonstrate the level of detail achieved by vis-OCTF, Miller et al.^55^ compared the montaged vis-OCT fibergrams with their corresponding confocal images of immunostained flat-mounted retinas to indicate RGC axons, as illustrated in Fig. 7. The orange arrows (labeled 1 to 4) highlighted four small axon bundles visible in both vis-OCTF and confocal microscopy corresponding to variable diameters, and the red arrows (labeled 5 to 7) highlighted blood vessels visible in vis-OCTF but appeared as dark shadows in confocal microscopy. This result validated the capability of vis-OCTF to resolve individual RGC axon bundles with varying sizes *in vivo*.

## 4 Applications of vis-OCT

### 4.1 Applications in Pre-Clinical Studies

In animal studies, vis-OCT facilitates detailed investigation of disease mechanisms, therapeutic responses, and developmental processes. As conventional NIR-OCT, vis-OCT is usually applied to retinal imaging. Due to the spectroscopic contrast within visible light range, most animal studies focused on hemodynamics.

#### 4.1.1 Retinal hemodynamics

Retinal hemodynamics describes blood flow rate, oxygen saturation (${\mathrm{sO}}_{2}$), vessel diameter, and oxygen metabolic rate. Studies have indicated that hemodynamic variations precede the onset of noticeable capillary dropout;^111^^–^^114^ thus, these changes, as potential biomarkers, can be valuable for early diagnosis. The measurement of ${\mathrm{sO}}_{2}$ by vis-OCT has been demonstrated on rats.^22^^,^^24^^,^^64^^,^^80^^,^^84^^,^^85^ For example, Pi et al.^64^ used vis-OCT oximetry to differentiate arteries from veins and observed a preponderance of arterioles in the superficial vascular plexus, whereas venules tend to drain blood from the deep capillary plexus. This vascular organization suggests a primarily serial pattern of blood flow transition from arteries to veins in a rat’s retinal circulation.^64^ Later, the same group adopted OCTA technology to calculate ${\mathrm{sO}}_{2}$ at the capillary level to better understand the retinal circulation under different conditions, including normoxia, hypoxia, and hyperoxia.^25^ They found that venous ${\mathrm{sO}}_{2}$ changed more dramatically from hypoxia to hyperoxia than arterial ${\mathrm{sO}}_{2}$, and compared with major vessels, the ${\mathrm{sO}}_{2}$ in capillaries changed less during inhaled oxygen regulation. On top of that, ${\mathrm{sO}}_{2}$ is also measured to observe the retinal response to various diseases. Chong et al.^22^ demonstrated that ${\mathrm{sO}}_{2}$ was reduced to zero while all oxyhemoglobin was converted to deoxyhemoglobin after cardiac arrest. In addition, Soetikno et al.^115^ compared inner retinal ${\mathrm{sO}}_{2}$ between normal rats and rats with oxygen-induced retinopathy (OIR), finding that the average arterial ${\mathrm{sO}}_{2}$ was higher in the control group, but there was no significant difference in average venous ${\mathrm{sO}}_{2}$ between the two groups. Other responses include that venous ${\mathrm{sO}}_{2}$ showed a gradual decrease despite steady arterial ${\mathrm{sO}}_{2}$ as IOP is elevated,^84^ and ${\mathrm{sO}}_{2}$ in arteries and veins was significantly reduced at 4 weeks after optic nerve transection. ^85^

**Fig. 8:**
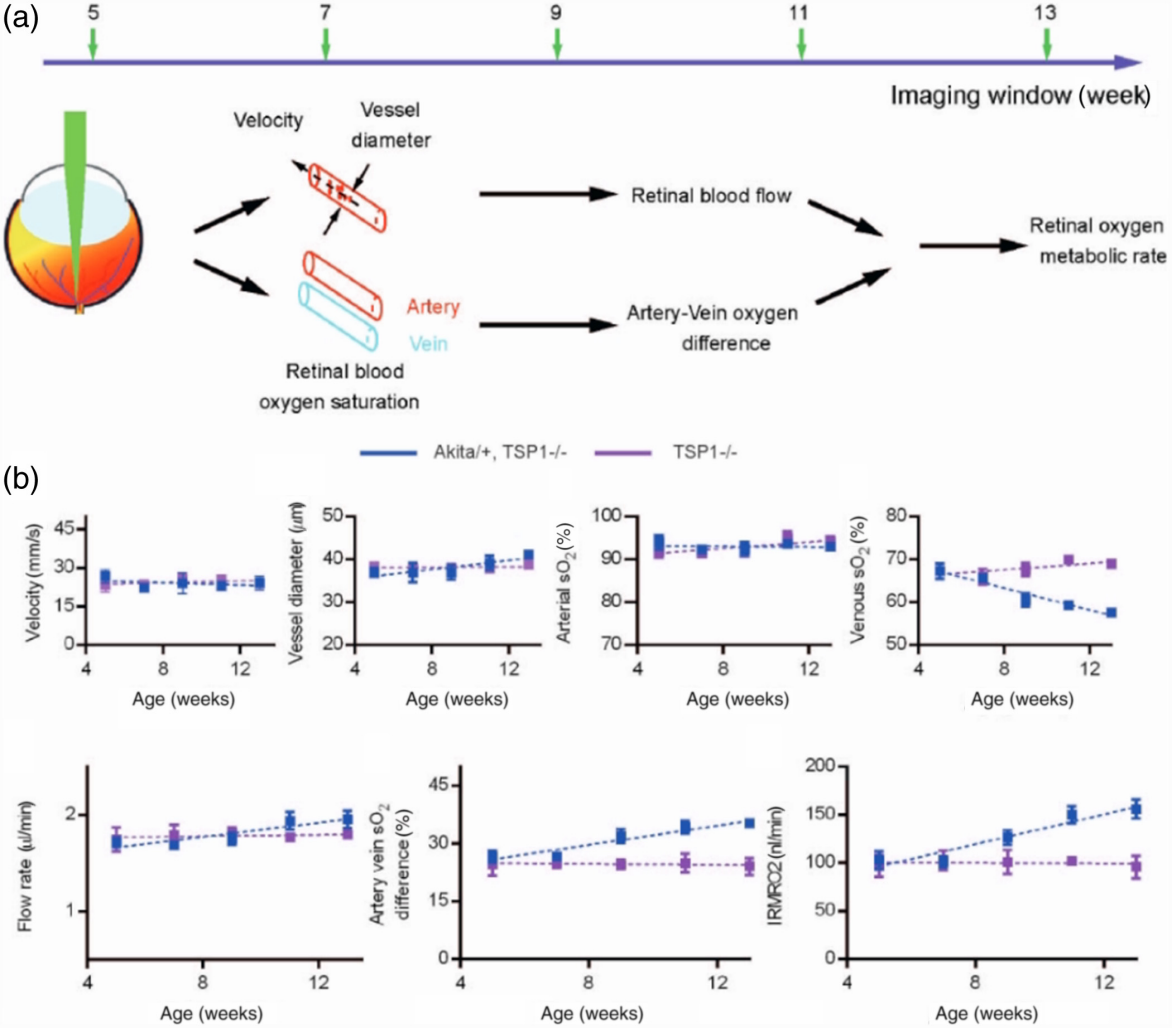
Longitudinal monitoring retinal flow, ${\mathrm{sO}}_{2}$, and ${\mathrm{rMRO}}_{2}$ for both DR mice (Akita/+, TSP1-/−) and control mice (TSP1−/−) from 5 to 13 weeks of age. (a) Schematic of retinal oxygen metabolism measurement. (b) Longitudinal monitoring results of mean retinal arterial blood velocity. (c) Longitudinal monitoring results of mean retinal arterial vessel diameter. (d) Longitudinal monitoring results of mean retinal arterial ${\mathrm{sO}}_{2}$. (e) Longitudinal monitoring results of mean retinal venous ${\mathrm{sO}}_{2}$. (f) Longitudinal monitoring results of mean retinal blood flow. (g) Longitudinal monitoring results of artery-vein ${\mathrm{sO}}_{2}$ difference. (h) Longitudinal monitoring results of ${\mathrm{rMRO}}_{2}$. Reproduced with permission from Ref. 114.

Another crucial hemodynamic parameter is retinal oxygen metabolic rate (${\mathrm{rMRO}}_{2}$), defined as oxygen consumption of the tissue over time:^64^

$${\mathrm{rMRO}}_{2}=\frac{4W_{O_{2}}}{W_{{\mathrm{HbO}}_{2}}}\cdot C_{\mathrm{HbT}}\cdot (\underset{i}{\sum }{\mathrm{sO}}_{2,a}F_{a,i}-\underset{i}{\sum }{\mathrm{sO}}_{2,v}F_{a,v})$$ (11)

where $W_{O_{2}}$ and $W_{{\mathrm{HbO}}_{2}}$ are the molecular weights of $O_{2}$ and oxygenated hemoglobin, $C_{\mathrm{HbT}}$ is the total hemoglobin concentration in blood, and ${\mathrm{sO}}_{2}$ and F are the measured oxygen saturation and blood flow in each vessel, respectively. Combining with Doppler OCT analysis, Pi et al.^64^ found that ${\mathrm{rMRO}}_{2}$ of rats in hyperoxic conditions were equivalent to that in normoxia and lower than that in hypoxic conditions. In a follow-up study,^84^ they conducted acute IOP elevation protocols on rats and observed that at IOPs of 50 and 60 mmHg, the arterial blood flow reversal began to appear in some vessels, resulting in reduced ${\mathrm{rMRO}}_{2}$. Similar strategies were reported on OIR rat models that closely mimic the exposure and findings of retinopathy of prematurity (ROP).^115^ Compared with normal controls, a 59% decrease in ${\mathrm{rMRO}}_{2}$ of the OIR group was observed on postnatal day 18, which might be caused by the declining neuronal oxygen utilization. In addition, Liu et al.^114^ used type-1 diabetic mice as the model of early-stage DR to investigate the hemodynamic variations. The results showed that during the observation period, the venous ${\mathrm{sO}}_{2}$ of diabetic mice decreased with age, leading to a significant increment (41.3%) in ${\mathrm{rMRO}}_{2}$. By contrast, other parameters, such as blood flow rate, vessel diameter, and arterial ${\mathrm{sO}}_{2}$ stayed approximately constant (Fig. 8). The histologic examination manifested no anatomical retinal alternations in both groups, which implied complications in retinal oxygen metabolism may occur before retinal structure change in DR.

#### 4.1.2 Retinal neuronal and vascular tissues

**Fig. 9:**
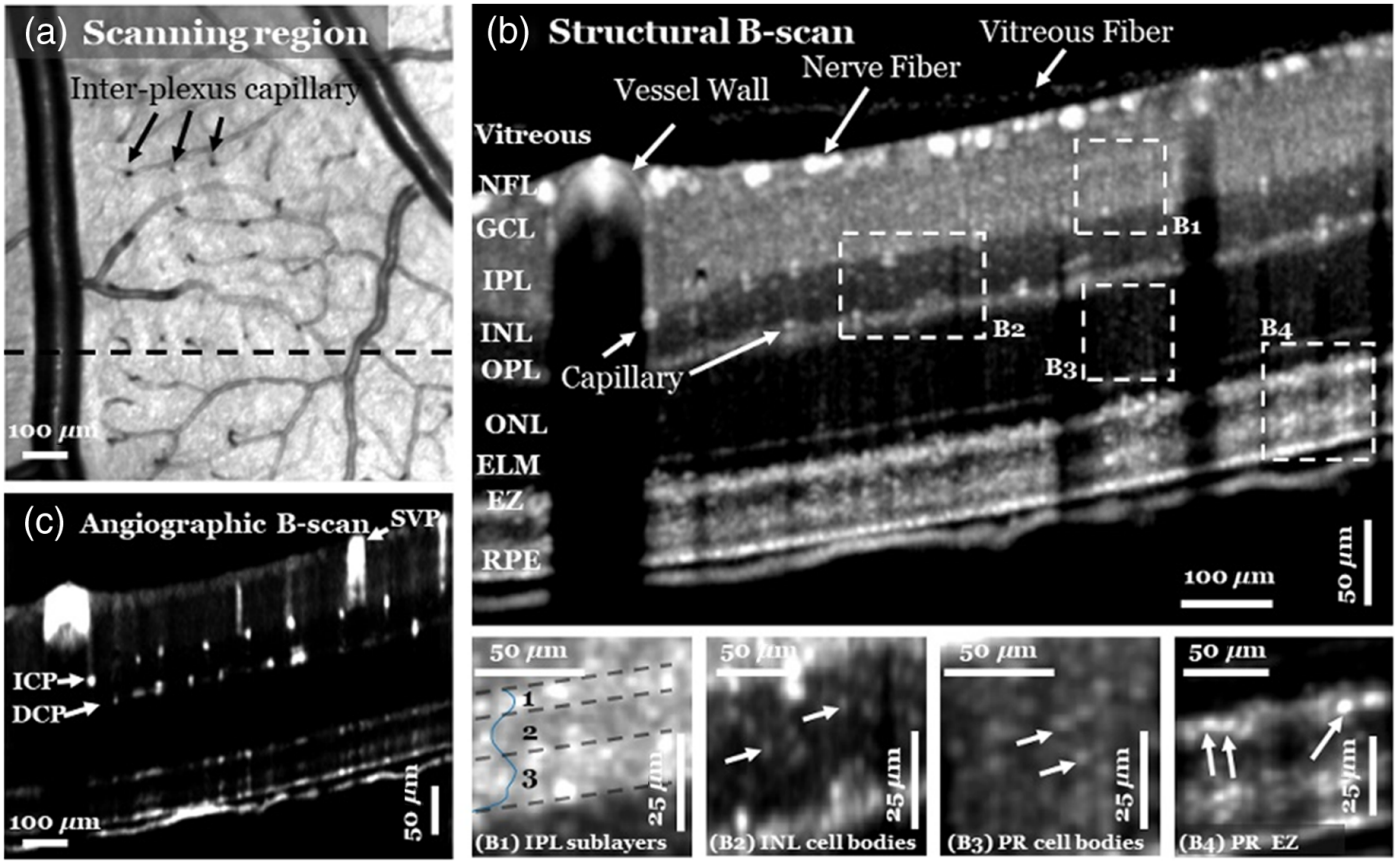
(a) Mean *en face* projection structural vis-OCT of a rat retina with $1\times 1{\;\mathrm{mm}}^{2}$ FOV from an averaged volume scan (N=110). Inter-plexus capillaries (arrows) can be recognized as dark dots. The black-dashed line indicates the location of the B-scan shown in panels (b) and (c). (b) Structural B-scan from the averaged volume visualizes (B1) at least three sub-laminar layers in the IPL, (B2) INL cell bodies, photoreceptor (PR) (B3) cell bodies, and (B4) EZ. The blue curve in (B1) is the averaged structural depth profile of the IPL. The white arrows indicate the representative neuronal cells. (c) Corresponding angiographic B-scan demonstrates large vessels and single capillaries. The flow signals in the outer retina are due to projection artifacts. SVP, superficial vascular plexus; ICP, intermediate capillary plexus; DCP, deep capillary plexus. Reproduced with permission from Ref. 116.

vis-OCT is also applied for other eye imaging purposes, which can be roughly categorized into two types. One is for structural information because it can provide better axial resolution than NIR-OCT. An example is that Pi et al.^116^ used vis-OCT with volumetric registration and averaging to achieve cellular-level retinal structural imaging in a rat eye, and they successfully visualized cells in the inner nuclear layer and photoreceptors in the outer nuclear layer and ellipsoid zone (Fig. 9). In addition, vis-OCT can also be used to retrieve the distribution of rhodopsin, the light-sensing molecule in the outer segments of rod photoreceptors, and thus assess the distribution and density of functional rod photoreceptors in the retina.^117^ Other than retinal imaging, Zhang et al.^118^ developed an anterior segment vis-OCT system and a compound circumlimbal scanning method to image the full Schlemm’s canal and its surrounding limbal vascular network in mice. They also visualized Schlemm’s canal size variations along the canal (parallel view) and perpendicular to the canal (perpendicular view) under different IOP levels and found that in both views, Schlemm’s canal cross-sectional areas were obviously larger at a lower IOP and decreased in size as IOP increased.

**Fig. 10:**
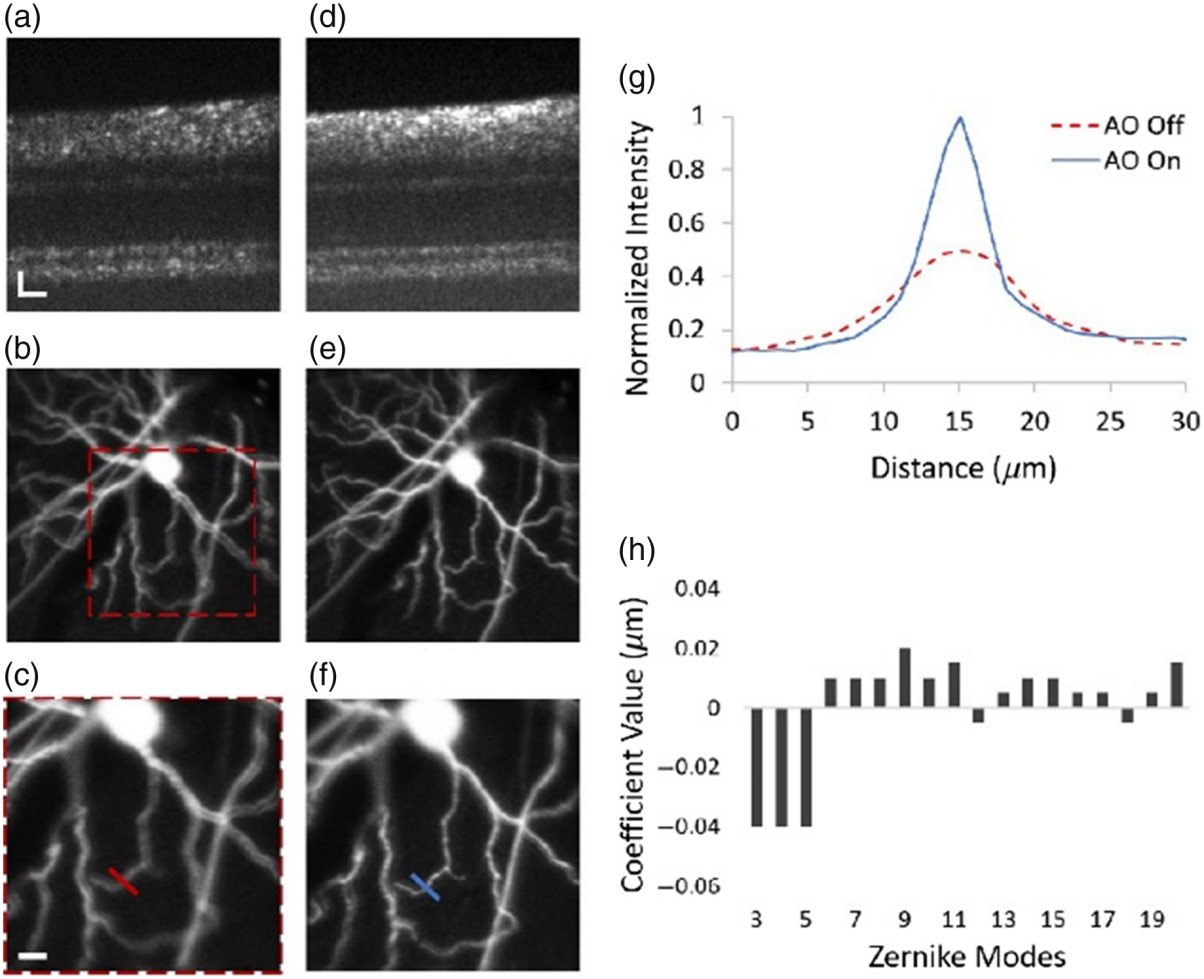
vis-OCT and fluorescence images before and after AO optimization. (a)–(c) OCT B-scan and fluorescence images of EGFP-labeled ganglion cells before optimization. (d)–(f) The corresponding optimized images by AO. (g) Line spread function taken across the red lines in panels (c) and (f), revealing the improvement in the lateral resolution after optimization. (h) Zernike coefficients selected during optimization. Reproduced with permission from Ref. 27.

The other type is functional imaging. vis-OCTA was reported to detect choroidal neovascularization,^119^ a hallmark of wet AMD subtype that may cause exudative leakage, hemorrhage, and irreversible vision loss,^120^ and the size of lesions could also be accurately measured. vis-OCTF can be applied to track the progression of retinal diseases and evaluate the effectiveness of potential treatments by measuring lateral width, axial height, cross-sectional area, and shape of the RGC axons.^109^^,^^110^ In addition, a large number of studies have worked on multimodal systems, achieving fluorescence imaging. These systems can be further divided depending on whether they use endogenous or exogenous fluorophores. A representative example of endogenous fluorophores is autofluorescence. Dai et al.^121^ integrated vis-OCT with autofluorescence imaging without requiring any additional light source. Using the simultaneously acquired OCT images as a reference, the effect of light attenuation caused by components anterior to RPE or RPE melanin can be eliminated, allowing for improved quantification of retinal autofluorescence intensity.^28^^,^^70^^,^^71^ Moreover, corrected autofluorescence signals could be used to assess the amount of lipofuscin in the RPE for diagnosis and monitoring disease progression.^122^^–^^124^ As for exogenous fluorophores, most studies used transgenic mice with EGFP-labeled cells. The aforementioned multimodal AO-OCT-SLO system,^27^ for example, imaged a mouse with ganglion cells labeled with EGFP. After the AO optimization, the lateral resolution and image quality for both modalities were apparently improved (Fig. 10). The same strategy could be used for other types of cells and features with different fluorophores, such as microglia cells.^125^^,^^126^

#### 4.1.3 Beyond the eye

**Fig. 11:**
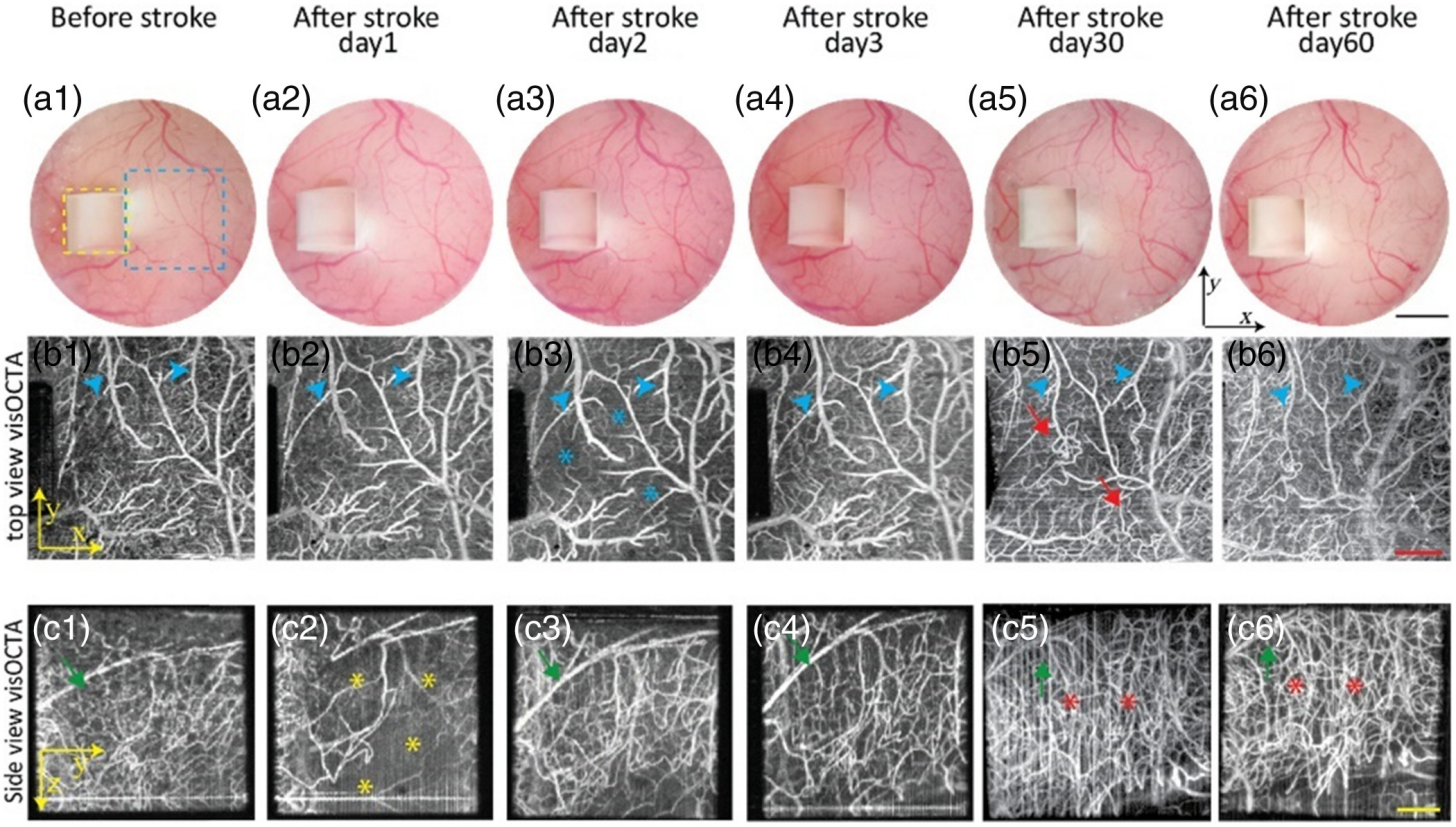
Longitudinal monitoring of acute (days 1 to 3) and chronic (days 30 and 60) changes after tMCAO. (a1)–(a6) Optical microscopic images from top-view before and after tMCAO. Yellow-dashed square: side view from the microprism. Blue-dashed square: top-view. (b1)–(b6) Top-view vis-OCTA *en face* images before and after tMCAO. Blue arrowheads: vessel dilation (b2)–(b4); vessel constriction (b5) and (b6). Blue stars: reduced flow signal. Red arrows: bounded neovascularization. (c1)–(c6) Side-view vis-OCTA *en face* images before and after tMCAO. Green arrows: vessel dilation (c3) and (c4); vessel narrowing (c5) and (c6). Yellow stars: reduced flow signal. Red stars: overgrown neovascularization. (Black scale bar, 1 mm; red scale bar, 400  μm, yellow scale bar, 200  μm). Reproduced with permission from Ref. 127.

In addition to the extensive application in eyes, vis-OCT has been used for other anatomical sites, such as the brain,^22^^,^^75^^,^^127^^,^^128^ skin,^107^ and female reproductive tract.^129^ For example, Chen et al.^128^ developed a novel dual-depth sampling and normalization strategy to improve the accuracy of vis-OCT oximetry in highly scattering mediums-brain cortex. They applied this approach to monitor the hemodynamic response in the mouse cortex after focal photothrombosis. They found that in the penumbra region vessel dilatation was negatively correlated with the original vessel diameter, and the ${\mathrm{sO}}_{2}$ of vessels also dropped below the normal range after focal ischemia. Later, the same group integrated LSI and vis-OCT to reveal dynamic vascular responses in acute stages in a dMCAO mouse model.^75^ The results indicated that after dMCAO, there was no significant variation in ${\mathrm{sO}}_{2}$ levels in arteries and arterioles, whereas a location-dependent drop in ${\mathrm{sO}}_{2}$ was observed in veins and venules. And higher branch-order veins had larger drops in ${\mathrm{sO}}_{2}$ at the re-perfusion stage. Furthermore, they surgically implanted a microprism in the mouse brain, enabling vis-OCT to image the entire depth of the cortex.^127^ They monitored the ischemic strokes caused by transient middle cerebral artery occlusion (tMCAO) for up to 60 days and observed different microvascular responses at various cortical depths. As Fig. 11 indicates, major vessel diameters shrank in the chronic stage as compared with baseline prior to stroke and acute stage, but non-perfused regions were observed in both the superficial and deep cortex in this period. Besides, Chong et al.^130^ used vis-OCT with Doppler and spectroscopic capabilities to measure the cerebral metabolic rate of oxygen in the mouse brain. The results showed that despite the large variations in oxygen saturation, there were no major changes in oxygen consumption in response to small changes in the proportion of inspired oxygen or carbon dioxide.

Another potential clinical application is colposcopy. Before vis-OCT, fiber-based NIR-OCT has been reported for colposcopic imaging, including vaginal epithelium examination and vaginal product evaluation. To more clearly resolve the mucosal and epithelial interface and implement 3D imaging capability, Duan et al.^129^ proposed the first vis-OCT system and two probe designs for colposcopic application. The high-resolution images of macaque vaginal tracts demonstrated that vis-OCT can successfully identify both epithelium and connective layers, map the thickness of the epithelial layer, detect biopsy lesions, and potentially identify minor disruptions within the vaginal epithelium.

### 4.2 Applications in Clinical Studies

Human imaging needs to consider a few more things than animal imaging. First, laser safety should meet the requirements of the American National Standard for Safe Use of Lasers maximum permissible exposure limit, and the incident power should be much smaller when conducted within a long continuous exposure time. Second, the human retina is sensitive to visible light, which may pose a challenge for eye fixation and result in more motion artifacts during image acquisition. Therefore, a quick imaging protocol is essential to minimize patient discomfort and encourage cooperation. Third, it has been found that both flow and retinal metabolism change when the retina transitions from a dark environment to bright or flicker stimulation;^131^ thus, it is vital to ensure the patient’s eye is light-adapted before vis-OCT imaging when quantitative measurements such as blood flow are performed.

The first demonstration of human retinal imaging using vis-OCT was reported by Yi et al. in 2015,^20^ with SLO integrated by sharing the same optical path in the sample arm for alignment purposes. In the beginning, they operated in SLO mode, rapidly identifying the region of interest (ROI) and optimizing axial focusing. Then, they switched to OCT mode, scanning the same ROI and manually adjusting the position of the reference mirror, then started the acquisition. They also made a comparison of anatomical structures in vis-OCT and a commercially available NIR-OCT and found that the signal was slightly weaker in the inner retinal layers, but the visibility and contrast increased in the outer retina in vis-OCT. Similar strategies using other modalities for alignment were published by Shu et al.^21^ and Wang et al.,^63^ which used a chip camera for pupil monitoring and a NIR channel to detect the desired locations, respectively. To reduce the impact of eye motion, Zhang et al.^54^ developed a novel, Fourier transform-free, software axial motion tracking algorithm with a fast, magnetically actuated stage to maintain near-optimal axial resolution and sensitivity. They also implemented spatially dependent numerical dispersion compensation for the first time in the human eye *in vivo*. The results clearly revealed both IPL lamination and a clear hypo-reflective space between Bruch’s membrane and RPE. In addition, some groups have been able to utilize vis-OCT to analyze parameters related to glaucoma. Song et al.^132^ used a custom-designed dual-channel device to test whether reflectivity and spectroscopy of the peripapillary retinal nerve fiber layer (pRNFL) in vis-OCT are correlated with the severity of glaucoma. They found that the vis-OCT pRNFL reflectivity was more sensitive in separating suspect/preperimetric glaucoma from normal eyes than NIR-OCT. And Ghassabi et al.^133^ adopted a vis-OCT system to investigate the difference in the IPL sublayer thickness between small cohorts of healthy and glaucomatous subjects. From imaged IPL sublayers, five reported morphological IPL strata were visualized, and the sublayer $L_{2}$ was found to play a major role in the IPL thinning in eyes with advanced glaucoma. Both these studies demonstrated that vis-OCT can provide reliable metrics for glaucoma assessment.

**Fig. 12:**
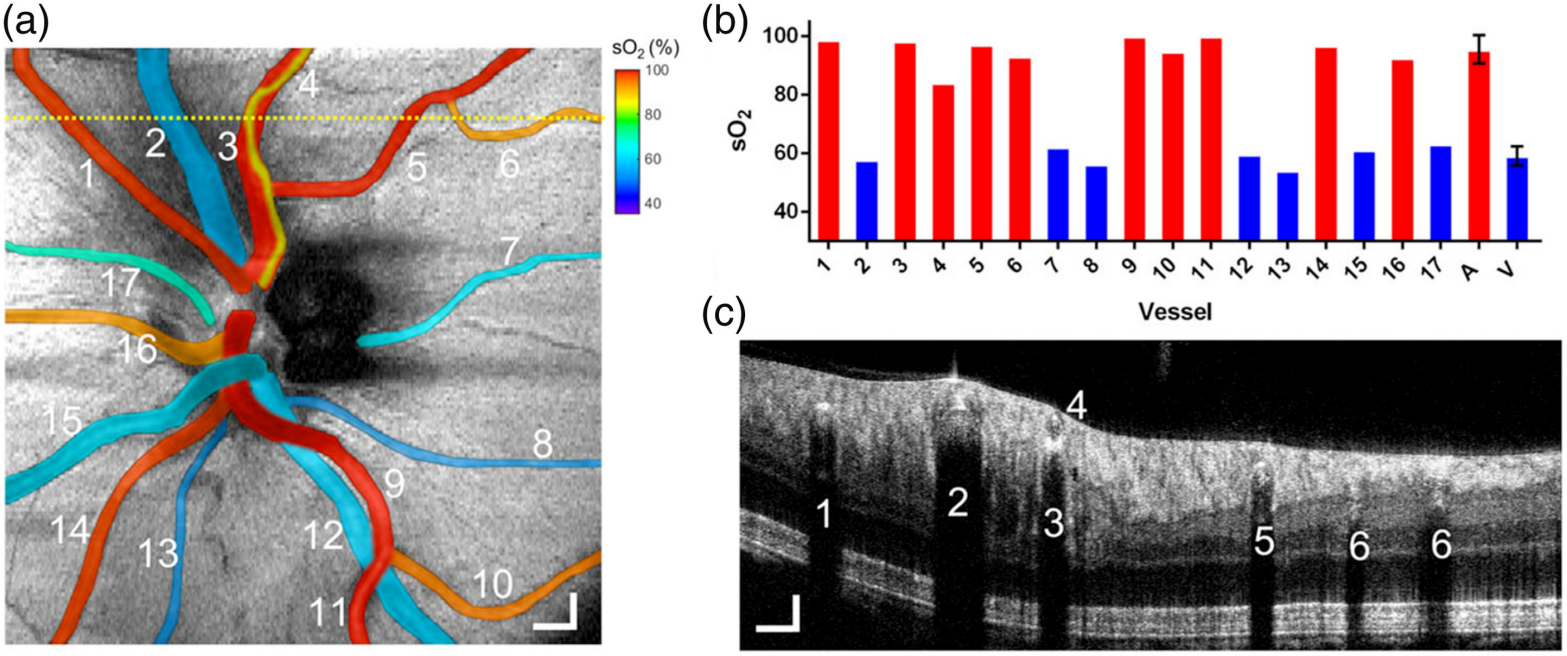
Oximetry map of the optic disk. (a) ${\mathrm{sO}}_{2}$ measurements in 17 vessels in the optic disk from a healthy 23-year-old. The ${\mathrm{sO}}_{2}$ values pseudo-colored and overlaid onto the fundus image. Scale bar: 300  μm. (b) Bar chart plots of ${\mathrm{sO}}_{2}$ measurements from panel (a) in individual arteries (red bar) and veins (blue bar) numbered from 1 to 17, as well as average ${\mathrm{sO}}_{2}$ in all arteries and all veins. (c) B-scan from the position highlighted by the yellow-dashed line in panel (a). Axially overlapping vessels in the retina require spectra contaminants removal, such as attenuation from vessel 4 in panel (c) would contaminate that in vessel 3. After the removal of spectra contaminants, the measured ${\mathrm{sO}}_{2}$ value in vessel 3 is 98.3%, consistent with the pulse oximeter reading (98%). Reproduced with permission from Ref. 134.

In addition, vis-OCT has been used for human retinal oximetry.^42^^,^^86^^,^^134^^,^^135^ To improve the accuracy of ${\mathrm{sO}}_{2}$ estimation when the signal-to-noise ratio (SNR) was low, Chen et al.^135^ developed a statistical-fitting-based sampling strategy, and the human experimental data verified the new approach reduced ${\mathrm{sO}}_{2}$ estimation error by 3 percentage points. Another effort to increase accuracy was reported by Soetikno et al.^86^ which repeatedly performed circumpapillary scans at the same location, with a combination of cross-correlation and graph-search-based segmentation to extract the vessel wall. This reduced the error and bias that may arise when averaging OCT signals along the length of a vessel. They found that there were diminishing benefits in OCT amplitude and ${\mathrm{sO}}_{2}$ accuracy after averaging ∼20 B-scans, but the exact number of averages may differ from various vis-OCT devices. Furthermore, Rubinoff et al.^134^ made a comprehensive analysis of unwanted signals that hampered vis-OCT’s clinical impact. These signals were referred to as spectra contaminants, which were increasingly magnified and unpredictable in human imaging compared with small animal imaging. In this work, they proposed adaptive spectroscopic vis-OCT, isolating blood’s oxygen-dependent spectrum by conforming measurements to the unique properties of each vessel, and 12 steps were involved for processing the retinal oximetry. The results have validated that this method can yield accurate and repeatable ${\mathrm{sO}}_{2}$ measurements in human retinal arteries and veins with varying diameters (Fig. 12).^134^ Aside from healthy subjects, vis-OCT oximetry has also been applied to routine patient imaging, such as in DR, central retinal vein occlusion, and sickle cell retinopathy.^125^ These studies confirm that functional information (angiography and ${\mathrm{sO}}_{2}$) provided by the vis-OCT system can potentially be used in the early detection and diagnosis of retinal pathologies, especially those ischemic in nature.^136^

vis-OCT has also been reported for imaging other tissues. For example, Revin et al.^69^ used it for the characterization of the skin barrier. In that work, images from the dorsal hand, ventral wrist, and ventral forearm areas were obtained, and the authors found that the appearance of the stratum corneum layer strongly depends on its water content, which became brighter after occlusive hydration. Cuartas-Vélez et al.^137^ demonstrated that the correlation of the vis-OCT signal can be used to visualize both spatial and temporal behavior of the thrombus formation in flowing human whole blood.

## 5 Prospects and Conclusions

vis-OCT has developed rapidly in the past few years. Compared with the counterpart NIR-OCT, it has higher axial resolution and higher scattering coefficients of biological tissue which increase imaging contrast. The ability to assess hemoglobin absorption facilitates quantitative oximetry, offering insight into oxygenation levels. Several techniques have been combined with vis-OCT for better image quality and functional imaging, including spectroscopic analysis,^23^ Doppler OCT,^64^ angiography,^24^ and AO.^27^ It could potentially benefit from other advanced developments for NIR-OCT as well.

Nevertheless, several challenges need to be addressed to fully realize the potential of vis-OCT. One of the primary objectives is the development of compact, cost-effective, and low-noise broadband light sources. Although several approaches have been reported to minimize the RIN and excess noise from the universally used SC light source, they are still not able to be eliminated thoroughly. Speckle noise, caused by self-interference of coherence light at random phases, is another source that reduces the image quality. A straightforward approach to suppressing speckle noise is to perform B-scan averaging. However, because eyes are sensitive to visible-light illumination, vis-OCT often suffers from increased eye motion. This makes B-scan averaging unreliable, even with image registration.^138^ Rubinoff et al.^138^ modulated both raster and circular scan protocols that enabled the acquisition of uncorrelated speckle patterns from similar anatomical locations to reduce the speckle noise. Ye et al.^139^ proposed a deep learning-based, end-to-end design for vis-OCT denoising. Therefore, with low-noise light sources and advanced noise removal technologies, vis-OCT is more likely to achieve the theoretical resolution and contrast in practice.

Additionally, because there is no available commercial visible light source for swept-source OCT (SSOCT) implementation, the maximum imaging speed of vis-OCT is limited by the line rate of the spectrometer, which cannot stack up against a NIR-SSOCT system. And increasing the exposure time of the camera to accumulate more photons reduces the speed even further. For clinical applications, long acquisition time under visible light exposure may result in patient discomfort and induce more motion artifacts; thus, the improvement of imaging speed might be the top priority for practical usage. Recently, the first swept-source vis-OCT (SS-vis-OCT) was demonstrated by Fan et al.,^140^ using a fanout periodically poled lithium niobate crystal for the second harmonic generation to convert a commercial NIR swept-source laser into a visible-light swept-source laser. Compared with spectrometer-based vis-OCT, SS-vis-OCT achieved a 3.3-fold increase in imaging depth, and the sensitivity roll-off improved to 0.5  dB/mm. However, the proposed visible-light swept-source laser needs further improvements, including output power and bandwidth.

Penetration depth is another hurdle. Due to the scattering and absorption properties, biomedical tissues tend to scatter and absorb more visible light than NIR light, leading to increased signal attenuation and degraded SNR. Therefore, since the choroidal circulation is beneath the highly optically absorbing and scattering RPE, it is challenging to resolve choroidal vessels and choriocapillaris because of the stronger attenuation. However, some strategies can be employed to enhance the SNR. Recently, Huang et al.^141^ proposed a deep-learning-based method, termed SNR-Net OCT, for low-light OCT images that have low brightness and SNR. This technology may be valuable in vis-OCT when details of deeper tissue are hard to visualize.

With these advanced technologies and algorithms, we expect that vis-OCT will play a more important role in the future. The predominant application will still be ophthalmology, and being deployed in the clinical setting for the diagnosis, development of disease progression, or treatment monitoring is the priority of these proof-of-concept techniques. It has been demonstrated that vis-OCT is a reliable tool to detect early variations of DR,^114^^,^^142^ ROP,^115^ AMD,^119^ glaucoma,^132^^,^^133^ and retinal vascular occlusive diseases^21^^,^^136^ through structural and functional information. Some of these findings are still at the animal research stage and translating them into clinical practice will be important. For routine patient ocular imaging, several factors need to be considered. First, the laser safety threshold of visible light is more strict than the NIR light, which is typically limited to around 0.2∼0.5  mW for continuous exposure.^21^ Second, the human eye is sensitive to visible light, posing a challenge to eye fixation during data acquisition, especially when the imaging speed is low. Real-time eye-tracking techniques can be incorporated into vis-OCT systems to minimize motion artifacts.^143^ Moreover, for widespread clinical applications, the cost is a big concern for commercialization. Low-cost visible light sources, such as low-repetition-rate supercontinuum sources along with balanced detection methods,^44^ multiple-SLD combined sources,^49^^,^^50^ and the emerging swept-source visible light laser^140^ could be potential choices. Undoubtedly, a cost-effective source without increasing system complexity is the most desirable.

Brain imaging is another primary clinical application in which vis-OCT is expected to play a role. Aside from hemodynamic investigation of healthy and diseased animal models, vis-OCT combined with endoscope design is able to image the deep brain with ultrahigh resolution.^68^ In addition, visible-light optical coherence microscopy, the transformation of OCT, has been reported to visualize the fine structures in the brain, including fibers,^144^^,^^145^ vascular structures,^144^^,^^145^ cell bodies,^145^ and amyloid plaques,^144^^,^^146^^–^^148^ which are a hallmark of Alzheimer’s disease.

To sum up, vis-OCT holds significant promise for advancing both biomedical research and clinical diagnostics due to its high resolution and ability to provide specific structural and functional information. Technological innovation, especially the light source, is crucial for the continued evolution and widespread adoption in healthcare. By combining state-of-the-art algorithms, such as deep learning networks, image quality and diagnostic accuracy could be improved. In the future, vis-OCT is expected to expand its applications across various medical fields, including ophthalmology, brain imaging, and dermatology.

## Data Availability

Data sharing is not applicable to this article, as no new data were created or analyzed.
